# Chronicles of nature calendar, a long-term and large-scale multitaxon database on phenology

**DOI:** 10.1038/s41597-020-0376-z

**Published:** 2020-02-11

**Authors:** Otso Ovaskainen, Evgeniy Meyke, Coong Lo, Gleb Tikhonov, Maria del Mar Delgado, Tomas Roslin, Eliezer Gurarie, Marina Abadonova, Ozodbek Abduraimov, Olga Adrianova, Tatiana Akimova, Muzhigit Akkiev, Aleksandr Ananin, Elena Andreeva, Natalia Andriychuk, Maxim Antipin, Konstantin Arzamascev, Svetlana Babina, Miroslav Babushkin, Oleg Bakin, Anna Barabancova, Inna Basilskaja, Nina Belova, Natalia Belyaeva, Tatjana Bespalova, Evgeniya Bisikalova, Anatoly Bobretsov, Vladimir Bobrov, Vadim Bobrovskyi, Elena Bochkareva, Gennady Bogdanov, Vladimir Bolshakov, Svetlana Bondarchuk, Evgeniya Bukharova, Alena Butunina, Yuri Buyvolov, Anna Buyvolova, Yuri Bykov, Elena Chakhireva, Olga Chashchina, Nadezhda Cherenkova, Sergej Chistjakov, Svetlana Chuhontseva, Evgeniy A. Davydov, Viktor Demchenko, Elena Diadicheva, Aleksandr Dobrolyubov, Ludmila Dostoyevskaya, Svetlana Drovnina, Zoya Drozdova, Akynaly Dubanaev, Yuriy Dubrovsky, Sergey Elsukov, Lidia Epova, Olga S Ermakova, Olga Ermakova, Aleksandra Esengeldenova, Oleg Evstigneev, Irina Fedchenko, Violetta Fedotova, Tatiana Filatova, Sergey Gashev, Anatoliy Gavrilov, Irina Gaydysh, Dmitrij Golovcov, Nadezhda Goncharova, Elena Gorbunova, Tatyana Gordeeva, Vitaly Grishchenko, Ludmila Gromyko, Vladimir Hohryakov, Alexander Hritankov, Elena Ignatenko, Svetlana Igosheva, Uliya Ivanova, Natalya Ivanova, Yury Kalinkin, Evgeniya Kaygorodova, Fedor Kazansky, Darya Kiseleva, Anastasia Knorre, Leonid Kolpashikov, Evgenii Korobov, Helen Korolyova, Natalia Korotkikh, Gennadiy Kosenkov, Sergey Kossenko, Elvira Kotlugalyamova, Evgeny Kozlovsky, Vladimir Kozsheechkin, Alla Kozurak, Irina Kozyr, Aleksandra Krasnopevtseva, Sergey Kruglikov, Olga Kuberskaya, Aleksey Kudryavtsev, Elena Kulebyakina, Yuliia Kulsha, Margarita Kupriyanova, Murad Kurbanbagamaev, Anatoliy Kutenkov, Nadezhda Kutenkova, Nadezhda Kuyantseva, Andrey Kuznetsov, Evgeniy Larin, Pavel Lebedev, Kirill Litvinov, Natalia Luzhkova, Azizbek Mahmudov, Lidiya Makovkina, Viktor Mamontov, Svetlana Mayorova, Irina Megalinskaja, Artur Meydus, Aleksandr Minin, Oleg Mitrofanov, Mykhailo Motruk, Aleksandr Myslenkov, Nina Nasonova, Natalia Nemtseva, Irina Nesterova, Tamara Nezdoliy, Tatyana Niroda, Tatiana Novikova, Darya Panicheva, Alexey Pavlov, Klara Pavlova, Polina Petrenko, Sergei Podolski, Natalja Polikarpova, Tatiana Polyanskaya, Igor Pospelov, Elena Pospelova, Ilya Prokhorov, Irina Prokosheva, Lyudmila Puchnina, Ivan Putrashyk, Julia Raiskaya, Yuri Rozhkov, Olga Rozhkova, Marina Rudenko, Irina Rybnikova, Svetlana Rykova, Miroslava Sahnevich, Alexander Samoylov, Valeri Sanko, Inna Sapelnikova, Sergei Sazonov, Zoya Selyunina, Ksenia Shalaeva, Maksim Shashkov, Anatoliy Shcherbakov, Vasyl Shevchyk, Sergej Shubin, Elena Shujskaja, Rustam Sibgatullin, Natalia Sikkila, Elena Sitnikova, Andrei Sivkov, Nataliya Skok, Svetlana Skorokhodova, Elena Smirnova, Galina Sokolova, Vladimir Sopin, Yurii Spasovski, Sergei Stepanov, Vitalіy Stratiy, Violetta Strekalovskaya, Alexander Sukhov, Guzalya Suleymanova, Lilija Sultangareeva, Viktorija Teleganova, Viktor Teplov, Valentina Teplova, Tatiana Tertitsa, Vladislav Timoshkin, Dmitry Tirski, Andrej Tolmachev, Aleksey Tomilin, Ludmila Tselishcheva, Mirabdulla Turgunov, Yurij Tyukh, Van Vladimir, Elena Vargot, Aleksander Vasin, Aleksandra Vasina, Anatoliy Vekliuk, Lidia Vetchinnikova, Vladislav Vinogradov, Nikolay Volodchenkov, Inna Voloshina, Tura Xoliqov, Eugenia Yablonovska-Grishchenko, Vladimir Yakovlev, Marina Yakovleva, Oksana Yantser, Yurij Yarema, Andrey Zahvatov, Valery Zakharov, Nicolay Zelenetskiy, Anatolii Zheltukhin, Tatyana Zubina, Juri Kurhinen

**Affiliations:** 1 University of Helsinki, PO BOX 65, 00014 Helsinki, Finland; 2 Centre for Biodiversity Dynamics, Department of Biology, Norwegian University of Science and Technology, N-7491 Trondheim, Norway; 3 EarthCape OY, Latokartanonkaari 3, 00790 Helsinki, Finland; 4 Oviedo University, Research Unit of Biodiversity (UMIB, UO-CSIC-PA), Campus Mieres, 33600 Mieres, Spain; 5 Swedish University of Agricultural Sciences, Department of Ecology, PO BOX 7044, SE-75007 Uppsala, Sweden; 6 University of Maryland, 3237 Biology-Psychology Building, University of Maryland, College Park, MD 20742 United States; 7 National Park Orlovskoe Polesie, 303943 Orel region Hotynetskiy district, Zhuderskiy village, Shkolnaya st. 2, Russian Federation; 8 Institute of Botany, Academy of sciences of the Republic of Uzbekistan, 100053 Tashkent, Bogi shamol str. 232 V, Uzbekistan; 9 Kostomuksha Nature Reserve, 186930 Karelia Republic Kostomuksha, Priozernaya 2, Russian Federation; 10 Altai State Nature Biosphere Reserve, 649000 Altai Republic Gorno-Altaysk, Naberezhnyi st., 1, Russian Federation; 11 Kabardino-Balkarski Nature Reserve, 360000 Kabardino-Balkaria Cherek District, Mechieva 78, Russian Federation; 12 FSE Zapovednoe Podlemorye, 671623 Republic of Buryatia Ust-Bargizin, Lenina st. 71, Russian Federation; 13 State Nature Reserve Stolby, 660006 Krasnoyarsk region Krasnoyarsk, Kariernaya 26, Russian Federation; 14 Carpathian Biosphere Reserve, 90600 Zakarpatska obl. Rakhiv, Krasne Pleso Str. 77, Ukraine; 15 Nizhne-Svirsky State Nature Reserve, 18700 Leningrad Region Lodeinoe Pole, Svir River, 1, Russian Federation; 16 State Nature Reserve Prisursky, 428034 Cheboksary, Lesnoj, 9, Russian Federation; 17 Zapovednoe Pribajkalje (Bajkalo-Lensky State Nature Reserve, Pribajkalsky National Park), 664050 Irkutsk, Bajkalskaya St., 291B, Russian Federation; 18 Darwin Nature Biosphere Reserve, 162723 Cherepovets District, Vologda Region Borok, 44, p/o Ploskovo, Russian Federation; 19 Volzhsko-Kamsky National Nature Biosphere Rezerve, 422537 Tatarstan Republic Zelenodolsk District, p/o Raifa, Sadovy, str. Vechova, 1, Russian Federation; 20 FGBU National Park Shushenskiy Bor, 662710 Krasnoyarsk Region Shushenskoe, Lugovaja 9, Russian Federation; 21 Voronezhsky Nature Biosphere Reserve, 394080, Centralnaja usadba, Goszapovednik, Voronezh, Russian Federation; 22 Baikalsky State Nature Biosphere Reserve, 671220 Buryatia Republic Kabansky District, Tankhoy, 34 Krasnogvardeyskaya Street, Russian Federation; 23 Visimsky Nature Biosphere Reserve, 624140 Kirovgrad, Stepana Razina, 23, Russian Federation; 24 Kondinskie Lakes National Park named after L. F. Stashkevich, 628240, Hanty-Mansijsk district, City Sovietsky, Komsomolski st., 5, Russian Federation; 25 Federal State Organization of Joint Direction of Kedrovaya Pad’ State Biosphere Nature Reserve and Leopard’s Land National Park, 690068 Primorskiy kray Vladivostok, pr. 100-letiya Vladivostoka 127, Russian Federation; 26 Pechoro-Ilych State Nature Reserve, 169436, Komi Republic, Trinity-Pechora region, Yaksha, Laninoy Street 8, Russian Federation; 27 A. N. Severtsov Institute of Ecology and Evolution, 119071 Moscow, Leninsky Prospect 33, Russian Federation; 28 FGBU Zapovednoye Priamurye, Komsomolskiy Department, 681000 Khabarovskyi krai Komsomolsk-on-Amur, Mira avenue, 54, Russian Federation; 29 Tigirek State Nature Reserve, 656043 Barnaul, Nikitina street 111, Russian Federation; 30 Institute of Systematics and Ecology of Animals of Siberian Branch of Russian Academy of Science, 930091 Novosibirsk, Frunze 11, Russian Federation; 31 State Nature Reserve Bolshaya Kokshaga, 424038 Mary El Republic Yoshkar-Ola, Voinov-Internacionalistov 26, Russian Federation; 32 Institute of Plant and Animal Ecology, Ural Branch, Russian Academy of Sciences, 620100 Ekaterinburg, 8 Marta 202/3, Russian Federation; 33 Sikhote-Alin State Nature Biosphere Reserve named after K. G. Abramov, 692150 Primorsky krai Terney, Partizanskaya 44, Russian Federation; 34 FSBI Prioksko-Terrasniy State Reserve, 142200 Moscow region Serpukhov district, Danky, Russian Federation; 35 National park Meshchera, 601501 Vladimir region Gus-Hrustalnyi, Internacionalnaya 111, Russian Federation; 36 Ilmensky State Nature Reserve, Russian Academy of Sciences, Urals Branch, 456317 Chelyabinskaya oblast Miass, Russian Federation; 37 FGBU National Park Kenozersky, 163000 Arkhangelsk, Embankment of the Northern Dvina, 78, Russian Federation; 38 FGBU GPZ Kologrivskij les im. M.G. Sinicina, 157440 Kostromskaja oblast’ Kologriv, Nekrasova 48, Russian Federation; 39 Altai State University, 656049 Lenin Ave. 61, Barnaul, Russian Federation; 40 Pryazovskyi National Nature Park, 72312 Zaporiz’ka oblast Melitopol, Interkulturna Street, 21/1, Ukraine; 41 State Nature Reserve Privolzhskaya Lesostep, 440031 Penza, Okruzhnaya 12-a, Russian Federation; 42 Komarov Botanical Institute of the Russian Academy of Sciences (BIN RAS), 197376 Saint Petersburg, Professora Popova 2, Russian Federation; 43 Sary-Chelek State Nature Reserve, 715705 Dzalal-Abad region, Aksu district, Arkyt village, Kyrgyzstan; 44 Institute for Evolutionary Ecology NAS Ukraine, 03143 Kiev, Lebedeva 37, Ukraine; 45 FGBU State Nature Reserve Kuznetsk Alatau, 652888 Kemerovo region, Mezhdurechensk, Shakhterev 33-1, Russian Federation; 46 Kerzhenskiy State Nature Biosphere Reserve, 603001 Nizhny Novgorod, Rozhdestvenskaya 23, Russian Federation; 47 Bryansk Forest Nature Reserve, 242180 Bryansk region Suzemka district, Nerussa St., Zapovednaya street, 2, Russian Federation; 48 Pinezhsky State Nature Reserve, 164610 Arhangel region Pinezkiy district, Pinega, Pervomayskaya street, 123 А, Russian Federation; 49 The Central Chernozem State Biosphere Nature Reserve named after Professor V.V. Alyokhin, 305528 Kurskiy region Kurskiy district, p/o Zapovednoe, Russian Federation; 50 Tyumen State University, 625043 Tyumen, Pirogova str., 3, Russian Federation; 51 Reserves of Taimyr, 666300 Norilsk, str. Talnakhskaya, entrance 2, Russian Federation; 52 Chatkalski National Park, 100059 Toshkent, Shota Rustaveli St., 144-34, Uzbekistan; 53 National Park Ugra, 248007 Kaluga, Prigorodnoe lesnichestvo, 3a, Russian Federation; 54 Kaniv Nature Reserve, 19000 Kaniv, Shevchenko str. 108, Ukraine; 55 Smolenskoe Poozerje National Park, 216270 Smolensk Region Demidovskiy district, Przhevalskoe, Gurevitch street 19, Russian Federation; 56 FSBI Zeya State Nature Reserve, 676246 Stroitelnaya str. 71, Zeya, Amurskaya Oblast Russian Federation; 57 Polistovsky State Nature Reserve, 182840 Pskov region Bezhanitsy district, Bezhanitsy Sovetskaya street, 9B, Russian Federation; 58 Ural State Pedagogical University, 620017 Yekaterinburg, prosp. Kosmonavtov, 26, Russian Federation; 59 Institute of Mathematical Problems of Biology RAS – the Branch of the Keldysh Institute of Applied Mathematics of Russian Academy of Sciences, 142290 Moscow Region Pushchino, Prof. Vitkevicha 1, Russian Federation; 60 Kronotsky Federal Nature Biosphere Reserve, 684000 Kamchatka region Yelizovo, Ryabikova street 48, Russian Federation; 61 Zhiguli Nature Reserve, 445362 Samara region, P. Bakhilova Polyana, Zhigulyovskaya 1, Russian Federation; 62 Institute for Ecology and Geography, Siberian Federal University, 660041 Krasnoyarsk, 79 Svobodny pr., Russian Federation; 63 Central Forest State Nature Biosphere Reserve, 172521 Tver region Nelidovo district, Zapovedniy village, Russian Federation; 64 National Park Bashkirija, 453870 Bashkortostan Republic Meleuzovskiy district, Nurgush, Abubakirova 1, Russian Federation; 65 State Nature Reserve Kurilsky, 694500 Sakhalin Juzhno-Kurilsk, Zarechnaya 5, Russian Federation; 66 Vodlozersky National Park, 185002 Karelia Petrozavodsk, Parkovaya 44, Russian Federation; 67 State Nature Reserve Kivach, 186220 Kondopoga District, Republic of Karelia Russian Federation; 68 South-Ural Federal University, 4563304 Chelyabinskaya oblast Miass, ul. Kalinina 37, Russian Federation; 69 Saint-Petersburg State Forest Technical University, 194021 St. Petersburg, Institutsky per. 5, 1-338-3, Russian Federation; 70 Astrakhan Biosphere Reserve, 414021 Astrakhan, Tsaerv River Bank 119, Russian Federation; 71 FSBI United Administration of the Lazovsky State Reserve and national park Zov Tigra, 692980 Primorskiy Krai Lazovskiy District, Lazo, Centralnaya, 56, Russian Federation; 72 State Nature Reserve Tungusskiy, 660028 Krasnoyarsk region Krasnoyarsk, Street 27 19, Russian Federation; 73 Krasnoyarsk State Pedagogical University named after V.P. Astafyev, 660049 Krasnoyarsk, Ada Lebedeva st. 89, Russian Federation; 74 Institute of Geography, Russian Academy of Sciences, 119017 Moscow, Staromonetniy 29, Russian Federation; 75 Koltzov Institute of Developmental Biology, Russian Academy of Sciences, 119334 Moscow, Vavilov Street 26, Russian Federation; 76 Carpathian National Nature Park, 78500 Ivano-Frankivsk region Yaremche, V. Stusa street 6, Ukraine; 77 State Environmental Institution National Park Braslav lakes, 211970 Vitebsk region Braslav, Dachnaya 1, Belarus; 78 National Park Synevyr, 90041 Zakarpattia Region Mizhhirs’kyi district, Synevyr-Ostriki, Ukraine; 79 Pasvik State Nature Reserve, 184421 Murmansk region Nikel, Gvardeyskiy Ave. 43, Russian Federation; 80 Mari Chodra National Park, 425090 Mari El Republic Zvenigovsky District, Krasnogorsky Settlement, Tsentralnaya Street, 73, Russian Federation; 81 Information-Analytical Centre for Protected Areas, 123242 Moscow, Kapranova side-street 3, Russian Federation; 82 State Nature Reserve Vishersky, 618590 Perm region Krasnovishersk, Gagarina street 36B, Russian Federation; 83 State Nature Reserve Olekminsky, 678100 Republic Sakha Olekminsk, Filatova 6, Russian Federation; 84 Crimea Nature Reserve, 298514 Alushta, Partizanskaya, 42, Republic of Crimea; 85 Forest Research Institute Karelian Research Centre Russian Academy of Sciences, 185910 Karelia Petrozavodsk, Pushkinskaya 11, Russian Federation; 86 Black Sea Biosphere Reserve, 75600 Khersons’ka oblast Hola Prystan, Mikhail Lermontov 1, Ukraine; 87 Institute of Physicochemical and Biological Problems in Soil Sciences, Russian Academy of Science, 142290 Moscow Region Pushchino, Institutskaya 2, Russian Federation; 88 State Nature Reserve Nurgush, 610002 Kirov, Lenina street, 129a, Russian Federation; 89 Caucasian State Biosphere Reserve of the Ministry of Natural Resources, 385000 Adygea Republik Maykop, Sovetskaya str. 187, Russian Federation; 90 National Nature Park Vyzhnytskiy, 59200 Chernivtsi Region Vyzhnytsya District, Berehomet, Street Central 27 а, Ukraine; 91 National Park Khvalynsky, 412780 Region Saratov Khvalynsk Sity, Oktyberskya Street, 2b, Russian Federation; 92 State Research Center Arctic and Antarctic Research Institute, 199397 Saint Petersburg, Bering st. 38, Russian Federation; 93 Mordovia State Nature Reserve, 431230 Mordovia Republic Temnikov region, village Pushta, Russian Federation; 94 State Nature Reserve Malaya Sosva, 628242 Tjumen region Sovetskiy, Lenina str., 46, Russian Federation; 95 Surhanskiy State Nature Reserve, 191404 Surhandarja region Sherabad, Agahi, 1, Uzbekistan

**Keywords:** Biodiversity, Phenology

## Abstract

We present an extensive, large-scale, long-term and multitaxon database on phenological and climatic variation, involving 506,186 observation dates acquired in 471 localities in Russian Federation, Ukraine, Uzbekistan, Belarus and Kyrgyzstan. The data cover the period 1890–2018, with 96% of the data being from 1960 onwards. The database is rich in plants, birds and climatic events, but also includes insects, amphibians, reptiles and fungi. The database includes multiple events per species, such as the onset days of leaf unfolding and leaf fall for plants, and the days for first spring and last autumn occurrences for birds. The data were acquired using standardized methods by permanent staff of national parks and nature reserves (87% of the data) and members of a phenological observation network (13% of the data). The database is valuable for exploring how species respond in their phenology to climate change. Large-scale analyses of spatial variation in phenological response can help to better predict the consequences of species and community responses to climate change.

## Abstract (summary)

**Table:** 

Measurement(s)	phenological event • climate
Technology Type(s)	digital curation
Factor Type(s)	geographic location • year
Sample Characteristic - Location	Russia • Ukraine • Uzbekistan • Belarus • Kyrgyzstan

Machine-accessible metadata file describing the reported data: 10.6084/m9.figshare.11687169

## Background & Summary

Phenological dynamics have been recognised as one of the most reliable bio-indicators of species responses to ongoing warming conditions^1^. Together with other adaptive mechanisms (e.g. changes in the spatial distribution and physiological adaptations), phenological change is a key mechanism by which plants and animals adapt to a changing world^2,3^. Many studies have documented that in the northern hemisphere, spring events have become earlier whereas autumn events are occurring later than before, mostly due to rising temperatures^4–6^. Despite this broadly shared response, there are systematic differences in phenological responses to climate change among individual species^7–9^, different taxonomic groups and trophic levels^10–12^. Further, while some studies have reported that different species are likely to have evolved distinct phenological responses to environmental cues^13,14^, others suggest that many species are synchronised because phenotypic plasticity in phenological response to climate may maintain local adaptation^15,16^.

Comprehensive understanding of phenological responses to climate change requires community-wide data that are both long-term and spatially extensive^11,17,18^. Such data are still not common and, with few exceptions^11,17,18^, the assessments of broad-scale taxonomic and geographic variations in phenological changes have generally involved meta-analyses^5,19^, or analyses of large observational databases that either represent mid-latitude systems^4,5,20^ or are characterized by low species richness^13^. Therefore, the spatial variation in phenological dynamics of species communities at large scale is still not well known^13,17^. Yet, this information is essential for understanding how species and communities respond to climate change^16^. A further common problem with many previously published data sets is publication bias. Few scientific journals are keen to publish papers reporting no detectable signal in species response to climate change – which can result in strongly biased conclusions in meta-analyses (but see^12,13^). Assembling monitoring data which has been consistently collected over long time and a large spatial extent addresses these problems directly^12^.

**Fig. 1:**
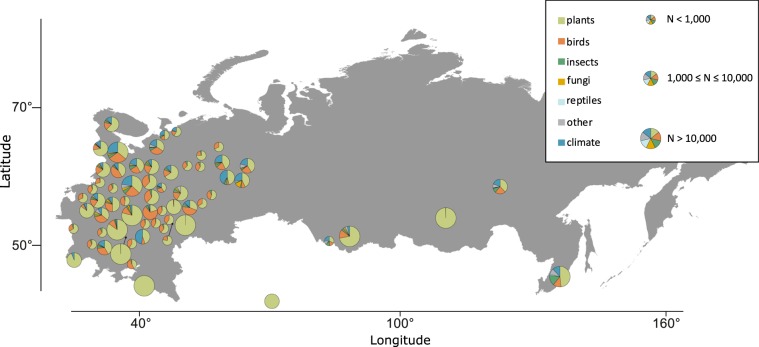
Spatial and taxonomic distribution of data. The size of each circle shows the total number of phenological observations, and the coloured sectors the proportions of observations belonging to each taxonomical group. The number of distinct localities in the database is 471, but in the figure data from nearby locations have been pooled into 63 locations which are situated at least 100 km apart.

**Fig. 2:**
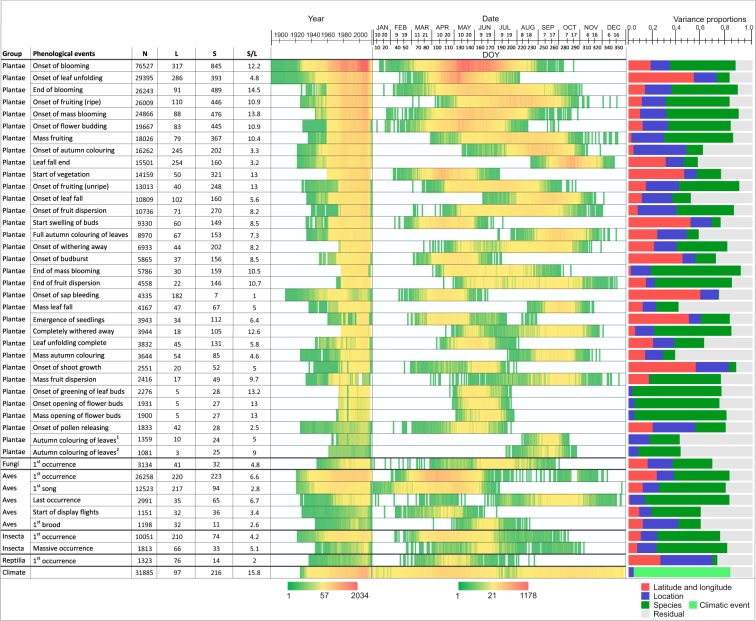
Illustration of the structure of the data for phenological events with highest coverage. Each row corresponds to a type of phenological event. For each event, shown are the total number of records (N), the number of locations from which the records originate (L), the number of species that the data involve (S), and the mean number of species per location (S/L). The two heat maps show the temporal coverage of data in terms of years included (reflecting data availability), and in terms of the phenological dates (reflecting the timing of the included events). Further shown is a variance partitioning, with the colours corresponding to the fixed effects of latitude, longitude and their interaction (red), the random effect of the site (blue), the random effect of the taxon or climatic parameter (green), and the residual (grey). The event types are ordered within each taxonomic group according to the total amount of data.

We present a large-scale and long-term dataset that can be used to examine community-level spatial variation in phenological dynamics and its climatic drivers. The database consists of 506,186 observation dates collected in 471 localities in the Russian Federation, Ukraine, Uzbekistan, Belarus and Kyrgyzstan (Fig. 1) over a 129-year period (from 1890 to 2018). During this period, researchers intensively conducted regular observations to record dates at which a predefined list of phenological and climatic events (Fig. 2) occurred. Although 96% of the observations were acquired from 1960 onwards, a few time series are very long. Events measured for plants include e.g. the onset days of leaf unfolding, first flowering time, and leaf fall; for birds they include e.g. days for first spring and last autumn occurrences; for insects, amphibians, reptiles and fungi they include e.g. day of first occurrence in the spring. The plant data were acquired in fixed plots, and the bird data along established routes. Climatic events were recorded as calendar dates when those events took place. Of all phenological dates, 87% were collected by research personnel of nature protected areas and national parks, who followed a systematic protocol. Thus, sampling effort remained nearly constant over time. The remaining 13% of the observations came from a well-established volunteer phenological network of volunteers, who followed a similar systematic protocol.

The recording scheme implemented at nature reserves offers unique opportunities for addressing community-level change across replicate local communities^21^. These data have been systematically collected not as independent monitoring efforts, but using a shared and carefully standardized protocol adapted for each local community. Thus, variability in observation effort is of much less concern than in most other distributed cross-taxon phenological monitoring schemes. To enable analyses of higher-level taxonomical groups, we have included taxonomic classifications for the species in the database.

## Methods

### Data acquisition

The data were collected by two research programs: the Chronicles of Nature (*Letopisi Prirody*) monitoring program, and a volunteer network of phenological observers (*Fenologicheskii Klub*). The Chronicles of Nature monitoring program^22^ is based on the network of strictly protected areas (zapovedniks) and national parks. The program gradually evolved during early 1900s^23^ and was formally established in 1940 with the aim of streamlining scientific work in protected areas with standardized methodology among the organizations. The program involves the permanent personnel of each participating organization. The results of the monitoring programs are published annually as Chronicle of Nature books. One printed copy of the books was kept in the office of the participating organization and another copy was sent to the Governmental Environmental Conservation Service (or a corresponding entity depending on the specific point in time).

In the Chronicles of Nature monitoring program, bird phenological events are extracted from route-based observations conducted regularly by ornithologists or professional rangers of the protected areas. Plant phenological events are reported either by botanists who visit permanent monitoring plots or transect, or by rangers who conduct regular walk-throughs within the strictly protected area or national park. The insect phenological data are extracted from standardized trapping data collected by entomologists on permanent plots or transects. The amphibian and reptile data are extracted from standardized trapping data collected by herpetologists. The fungal phenological data are collected by mycologists on permanent plots or transects. The weather event data are collected following a list of pre-defined events (e.g. first day of snowfall) by dedicated personnel or sourced from observations made on a local meteorological station. The types of data collected by each organization depends on the expertise of different taxonomic groups in the scientific personnel. For more details on how the data were collected, see^22,24–28^.

The network of phenological volunteer observers was established by the Russian Geographic Society in 1848 with questionnaires sent out to selected contacts among scientific community, including teachers and general public^29^. The participants of the volunteer observation network make observations throughout the year to collect data on a pre-defined limited set of phenological events related to plants, animals, and weather. The species included in the pre-defined lists were selected so that they could be identified reliably without specific taxonomical training.

### Data digitalization and unification

The compilation of the data in a common database was initiated in the context of the project “Linking environmental change to biodiversity change: long-term and large-scale data on European boreal forest biodiversity” (EBFB), funded for 2011–2015 by the Academy of Finland, and continued with the help of other funding to OO since 2016. We organized a series of project meetings that were essential for data acquisition, digitalization and unification. These meetings were organized in Ekaterinburg (Russia) by the Institute of Plant and Animal Ecology, Ural Branch of RAS (Russian Academy of Sciences) in 2011; in Petrozavodsk (Russia) by the Forest Research Institute, at the Karelian Research Center, RAS in 2013; in Miass (Russia) by the Ilmen Nature Reserve in 2014; in Krasnoyarsk (Russia) by the Stolby Nature Reserve in 2014; in Artybash (Russia) by the Altaisky Nature Reserve in 2015; in Listvyanka, Lake Baikal (Russia) by the Zapovednoe Pribajkalje Nature Reserve in 2016; in Roztochja (Ukraine) by the Ministry of Natural Resources of Ukraine in 2016; in Puschino (Russia) by the Prioksko-Terrasniy Nature Reserve in 2017, in Vyshinino (Russia) by the Kenozero National Park in 2018, and in St Petersburg (Russia) by the Komarov Botanical Institute of the Russian Academy of Sciences in 2019.

The compilation of the data into a common database was conducted by the database coordinators (EM and CL) in Helsinki (Finland). Those participants that already held the data in digital format submitted it in the original format, and those that had the data only in paper format digitized it using Excel-based templates developed in the project meetings. Submitted data were processed by the database coordinators according to the following steps:

1. The data were formatted so that each observation (the phenological date of a particular event in a particular locality and year) formed one row in the data table (e.g. un-pivoting tables that involved several years as the columns). The phenological event names were split into event type (e.g. “first occurrence“) and species name.
2. The event type names (provided originally typically in Russian) were translated into English and the species names (usually provided in Russian) were identified to scientific names, using dictionaries that were partly developed and verified in the project meetings. All scientific names were periodically verified by mapping them to the Global Biodiversity Information Facility (GBIF) backbone taxonomy^30^.
3. We associated each data record with the following set of information fields: (1) project name, i.e. the source organization, (2) dataset name, (3) locality name, (4) unique taxon identifier, (5) scientific taxon name, and (6) event type.
4. We imported the data records in the main database (maintained as an EarthCape database at https://ecn.ecdb.io). During the import, the taxonomic names, locality names, and dataset names were matched against already existing records.
5. The database was published in Zenodo^31^.

### Updates and limitations

There are at least 150 National Parks and Nature Reserves that collect Chronicles of Nature Book data (in Armenia, Azerbaijan, Belarus, Georgia, Kazakhstan, Kyrgyzstan, Moldova, Russian Federation, Tajikistan, Turkmenistan, Ukraine and Uzbekistan). Out of these, the current database covers data from 62 organizations, with the highest coverage in European Russia (Fig. 2). The collection of new data continues in most parks. Thus, the database is not complete, and we aim to support the database with updates, depending on the interest of new partners to join, as well as resources and funding. The technical validation procedures described below will also be applied to any new information included in the database. The resulting new versions of the database will be released through the Zenodo repository to ensure the long-term availability of the database.

The Chronicles of Nature programme involves several kinds of systematically collected data beyond phenology data: e.g. trapping data on small mammals, count data on birds, and yield data on berries and mushrooms^22^. We aim to publish these data as separate data papers.

## Data Records

The database is organized in six datasets: (1) a classification of taxa included, (2) a list of phenological events included, (3) a list of climatic events included, (4) information on the study site, (5) the phenology data, and (6) an information sources table for phenology data^31^. All tables are in csv format (columns separated by commas), and their fields are described in Tables 1–6. The tables can be linked to each other using the unique study site names and the unique identifiers for species and climatic evens.

**Table 1:** The fields of the taxonomy table (taxonomy.csv).

Field name	Description
taxonidentifier	The unique identifier of the taxon
taxon	The scientific name of the taxon
taxonomiclevel	The highest taxonomical level to which the taxon is described
kingdom	Kingdom
phylum	Phylum
class	Class
order	Order
family	Family
genus	Genus
species	Species
gbifkey	The GBIF key for the taxon
gbifstatus	Whether the GBIF status of the taxon is accepted

**Table 2:** The fields of the phenological events table (phenologicalevents.csv).

Field name	Description
kingdom	The kingdom for which the phenological event is relevant
eventtype	The name of the phenological event
description	The description of the phenological event
bbch	BBCH-scale used to identify the phenological development stages of plants^32^

**Table 3:** The fields of the climatic events table (climaticevents.csv).

Field name	Description
group	The type of the climatic event (e.g. related to temperature, snow or ice)
eventtype	The name of the climatic event
description	The description of the climatic event

**Table 4:** The fields of the study sites table (studysites.csv).

Field name	Description
studysite	The name of the study site
latitude	Latitude (typically of center of protected area)
longitude	Longitude (typically of center of protected area)

**Table 5:** The fields of the phenology table (phenology.csv).

Field name	Description
project	The name of the project in which the data were collected
dataset	The name of the dataset to which the data belongs to
studysite	The name of the study site in which the data were collected
taxonidentifier	The unique identifier of the taxon (“Climate” for climatic events)
taxon	The scientific name of taxon (climatic group for climatic events)
eventtype	The type of the event
year	The year
dayofyear	The date of the observation as the number of days since January 1^st^ in the focal year
quality	An indicator variable of any quality issues identified with the data

**Table 6:** The fields of the information sources table (informationsources.csv).

Field name	Description
project	The name of the project
source	The type of information sources (mostly Chronicles of Nature Books of the participating organizations)
reference	The references used to extract the phenology data

## Technical Validation

We asked the contributors to carefully check the validity of the phenological dates prior to submission. While uploading the submitted data to the database, we did manual validation checks to pinpoint data records that were suspicious (e.g. summer events recorded in winter), and sent the suspicious data records back for the contributors for correction or validation. However, given the extensive size of the database, it is likely that the database contains a number of erroneous records. Thus, we performed a series of checks to identify spurious data points and to examine for the strength of biological signal in the data.

First, we fitted for each (site – climatic/species name – event type) triplet a von Mises distribution, i.e. the circular normal distribution, where the circularity was used to connect the last day of the previous year to the first year of the next year. We identified as potentially spurious those records that were beyond the 0.9999367 central confidence interval of the fitted distribution (i.e. points located at least four standard deviations away from the mean, assuming a Gaussian distribution). This filtering revealed 322 severe outliers that were returned to the data owners for validation. If the data owner could neither verify nor correct the exceptional date, we marked this data record as suspicious.

Second, for each (site – climatic/species name – event type) triplet we fitted (i) a single von Mises distribution and (ii) a mixture of two von Mises distributions, and compared the fits of the models (i) and (ii) using the Bayesian Information Criteria (BIC). We identified the data as potentially spurious if the mixture model fitted better to the data with BIC difference of 5 or greater, and if the distance between the estimated means of the distributions in the mixture was greater than 30 days. For 214 such cases, we performed a manual examination of the data. This revealed e.g. the use of identical event names with different actual meaning (e.g. first arrival of the Willow Tit *Parus montanus*, recorded in spring and autumn seasons, and thus related to spring and autumn migration). Next, we repeated exactly the same filtering procedure, but for (climatic/species name – event type) pairs – to ensure that similarly named event types had consistent meaning across all sites.

### Major sources of variation in the data

To quantify the main sources of variation and thus to illustrate the types of ecological signals present in the data, we performed a variance partitioning analysis separately for each group of species and phenological events. As predictors, we used species and the location, the latter of which we further explained by the linear effects of latitude, longitude, and their interaction. These analyses were preformed using the LinearModelFit and Variance functions in Mathematica 11.1; Wolfram Research 2018. As an example, let us consider the event type with highest amount of data, which is the onset of blooming for plants. These data consist of 76,527 phenological dates, originating from 317 sites and representing 845 taxa (Fig. 2). We first computed for each site an average day over the species and years, resulting in 317 site-specific dates. These dates describe when plants on average (over years and plant species) have their onset of blooming on each location. While the collection of species included in the study varies from site to site, we still consider these dates meaningful proxies for the overall phenology of the onset of plant blooming. The amount of variation explained by the site-level averages was 33% of the original variance. Out of the variation explained by the site, 54% was further explained by the linear effects of latitude, longitude, and their interaction. We then partitioned the remaining variation (after the effect of site was accounted for) to components that could be attributed to the species (53% of the original variance) and to the residual (14% of the original variance). This analysis provided rather strong support for a strong ecological signal being present in the data, as 86% of the variation among the 76,527 data points could be attributed to the main effects of the location and species, and as ca. half of the variation among the locations could be attributed to a simple geographic trend. We note that the residual variation in this analyses should not be interpreted as erroneous noise, as it contains e.g. variation over time, and thus reflects e.g. the impact of climate change on phenology.

We repeated the above described analysis for all groups of phenological events for which there were at least 1000 data records, as well as climatic events related to temperature, snow, and ice. The results are illustrated in Fig. 2. The amount of explained variance is generally relatively high in all cases, suggesting that much of the variation in the data are explained by location and species.

## Data Availability

Not applicable.
