# Author Correction: Chronicles of nature calendar, a long-term and large-scale multitaxon database on phenology

**DOI:** 10.1038/s41597-020-0454-2

**Published:** 2020-04-09

**Authors:** Otso Ovaskainen, Evgeniy Meyke, Coong Lo, Gleb Tikhonov, Maria del Mar Delgado, Tomas Roslin, Eliezer Gurarie, Marina Abadonova, Ozodbek Abduraimov, Olga Adrianova, Tatiana Akimova, Muzhigit Akkiev, Aleksandr Ananin, Elena Andreeva, Natalia Andriychuk, Maxim Antipin, Konstantin Arzamascev, Svetlana Babina, Miroslav Babushkin, Oleg Bakin, Anna Barabancova, Inna Basilskaja, Nina Belova, Natalia Belyaeva, Tatjana Bespalova, Evgeniya Bisikalova, Anatoly Bobretsov, Vladimir Bobrov, Vadim Bobrovskyi, Elena Bochkareva, Gennady Bogdanov, Vladimir Bolshakov, Svetlana Bondarchuk, Evgeniya Bukharova, Alena Butunina, Yuri Buyvolov, Anna Buyvolova, Yuri Bykov, Elena Chakhireva, Olga Chashchina, Nadezhda Cherenkova, Sergej Chistjakov, Svetlana Chuhontseva, Evgeniy A. Davydov, Viktor Demchenko, Elena Diadicheva, Aleksandr Dobrolyubov, Ludmila Dostoyevskaya, Svetlana Drovnina, Zoya Drozdova, Akynaly Dubanaev, Yuriy Dubrovsky, Sergey Elsukov, Lidia Epova, Olga S Ermakova, Olga Ermakova, Aleksandra Esengeldenova, Oleg Evstigneev, Irina Fedchenko, Violetta Fedotova, Tatiana Filatova, Sergey Gashev, Anatoliy Gavrilov, Irina Gaydysh, Dmitrij Golovcov, Nadezhda Goncharova, Elena Gorbunova, Tatyana Gordeeva, Vitaly Grishchenko, Ludmila Gromyko, Vladimir Hohryakov, Alexander Hritankov, Elena Ignatenko, Svetlana Igosheva, Uliya Ivanova, Natalya Ivanova, Yury Kalinkin, Evgeniya Kaygorodova, Fedor Kazansky, Darya Kiseleva, Anastasia Knorre, Leonid Kolpashikov, Evgenii Korobov, Helen Korolyova, Natalia Korotkikh, Gennadiy Kosenkov, Sergey Kossenko, Elvira Kotlugalyamova, Evgeny Kozlovsky, Vladimir Kozsheechkin, Alla Kozurak, Irina Kozyr, Aleksandra Krasnopevtseva, Sergey Kruglikov, Olga Kuberskaya, Aleksey Kudryavtsev, Elena Kulebyakina, Yuliia Kulsha, Margarita Kupriyanova, Murad Kurbanbagamaev, Anatoliy Kutenkov, Nadezhda Kutenkova, Nadezhda Kuyantseva, Andrey Kuznetsov, Evgeniy Larin, Pavel Lebedev, Kirill Litvinov, Natalia Luzhkova, Azizbek Mahmudov, Lidiya Makovkina, Viktor Mamontov, Svetlana Mayorova, Irina Megalinskaja, Artur Meydus, Aleksandr Minin, Oleg Mitrofanov, Mykhailo Motruk, Aleksandr Myslenkov, Nina Nasonova, Natalia Nemtseva, Irina Nesterova, Tamara Nezdoliy, Tatyana Niroda, Tatiana Novikova, Darya Panicheva, Alexey Pavlov, Klara Pavlova, Polina Petrenko, Sergei Podolski, Natalja Polikarpova, Tatiana Polyanskaya, Igor Pospelov, Elena Pospelova, Ilya Prokhorov, Irina Prokosheva, Lyudmila Puchnina, Ivan Putrashyk, Julia Raiskaya, Yuri Rozhkov, Olga Rozhkova, Marina Rudenko, Irina Rybnikova, Svetlana Rykova, Miroslava Sahnevich, Alexander Samoylov, Valeri Sanko, Inna Sapelnikova, Sergei Sazonov, Zoya Selyunina, Ksenia Shalaeva, Maksim Shashkov, Anatoliy Shcherbakov, Vasyl Shevchyk, Sergej Shubin, Elena Shujskaja, Rustam Sibgatullin, Natalia Sikkila, Elena Sitnikova, Andrei Sivkov, Nataliya Skok, Svetlana Skorokhodova, Elena Smirnova, Galina Sokolova, Vladimir Sopin, Yurii Spasovski, Sergei Stepanov, Vitalіy Stratiy, Violetta Strekalovskaya, Alexander Sukhov, Guzalya Suleymanova, Lilija Sultangareeva, Viktorija Teleganova, Viktor Teplov, Valentina Teplova, Tatiana Tertitsa, Vladislav Timoshkin, Dmitry Tirski, Andrej Tolmachev, Aleksey Tomilin, Ludmila Tselishcheva, Mirabdulla Turgunov, Yurij Tyukh, Van Vladimir, Elena Vargot, Aleksander Vasin, Aleksandra Vasina, Anatoliy Vekliuk, Lidia Vetchinnikova, Vladislav Vinogradov, Nikolay Volodchenkov, Inna Voloshina, Tura Xoliqov, Eugenia Yablonovska-Grishchenko, Vladimir Yakovlev, Marina Yakovleva, Oksana Yantser, Yurij Yarema, Andrey Zahvatov, Valery Zakharov, Nicolay Zelenetskiy, Anatolii Zheltukhin, Tatyana Zubina, Juri Kurhinen

**Affiliations:** 1 University of Helsinki, PO BOX 65, 00014 Helsinki, Finland; 2 Centre for Biodiversity Dynamics, Department of Biology, Norwegian University of Science and Technology, N-7491 Trondheim, Norway; 3 EarthCape OY, Latokartanonkaari 3, 00790 Helsinki, Finland; 4 Oviedo University, Research Unit of Biodiversity (UMIB, UO-CSIC-PA), Campus Mieres, 33600 Mieres, Spain; 5 Swedish University of Agricultural Sciences, Department of Ecology, PO BOX 7044, SE-75007 Uppsala, Sweden; 6 University of Maryland, 3237 Biology-Psychology Building, University of Maryland, College Park, MD 20742 United States; 7 National Park Orlovskoe Polesie, 303943 Orel region Hotynetskiy district, Zhuderskiy village, Shkolnaya st. 2, Russian Federation; 8 Institute of Botany, Academy of sciences of the Republic of Uzbekistan, 100053 Tashkent, Bogi shamol str. 232 V, Uzbekistan; 9 Kostomuksha Nature Reserve, 186930 Karelia Republic Kostomuksha, Priozernaya 2, Russian Federation; 10 Altai State Nature Biosphere Reserve, 649000 Altai Republic Gorno-Altaysk, Naberezhnyi st., 1, Russian Federation; 11 Kabardino-Balkarski Nature Reserve, 360000 Kabardino-Balkaria Cherek District, Mechieva 78, Russian Federation; 12 FSE Zapovednoe Podlemorye, 671623 Republic of Buryatia Ust-Bargizin, Lenina st. 71, Russian Federation; 13 Institute of General and Experimental Biology, Siberian Department, Russian Academy of Sciences, 6, Sakhyanovoy str., Ulan-Ude, 670047 The Republic of Buryatia Russian Federation; 14 State Nature Reserve Stolby, 660006 Krasnoyarsk region Krasnoyarsk, Kariernaya 26, Russian Federation; 15 Carpathian Biosphere Reserve, 90600 Zakarpatska obl. Rakhiv, Krasne Pleso Str. 77, Ukraine; 16 Nizhne-Svirsky State Nature Reserve, 18700 Leningrad Region Lodeinoe Pole, Svir River, 1, Russian Federation; 17 State Nature Reserve Prisursky, 428034 Cheboksary, Lesnoj, 9, Russian Federation; 18 Zapovednoe Pribajkalje (Bajkalo-Lensky State Nature Reserve, Pribajkalsky National Park), 664050 Irkutsk, Bajkalskaya St., 291B, Russian Federation; 19 Darwin Nature Biosphere Reserve, 162723 Cherepovets District, Vologda Region Borok, 44, p/o Ploskovo, Russian Federation; 20 Volzhsko-Kamsky National Nature Biosphere Rezerve, 422537 Tatarstan Republic Zelenodolsk District, p/o Raifa, Sadovy, str. Vechova, 1, Russian Federation; 21 FGBU National Park Shushenskiy Bor, 662710 Krasnoyarsk Region Shushenskoe, Lugovaja 9, Russian Federation; 22 Voronezhsky Nature Biosphere Reserve, 394080, Centralnaja usadba, Goszapovednik, Voronezh, Russian Federation; 23 Baikalsky State Nature Biosphere Reserve, 671220 Buryatia Republic Kabansky District, Tankhoy, 34 Krasnogvardeyskaya Street, Russian Federation; 24 Visimsky Nature Biosphere Reserve, 624140 Kirovgrad, Stepana Razina, 23, Russian Federation; 25 Kondinskie Lakes National Park named after L. F. Stashkevich, 628240, Hanty-Mansijsk district, City Sovietsky, Komsomolski st., 5, Russian Federation; 26 Federal State Organization of Joint Direction of Kedrovaya Pad’ State Biosphere Nature Reserve and Leopard’s Land National Park, 690068 Primorskiy kray Vladivostok, pr. 100-letiya Vladivostoka 127, Russian Federation; 27 Pechoro-Ilych State Nature Reserve, 169436, Komi Republic, Trinity-Pechora region, Yaksha, Laninoy Street 8, Russian Federation; 28 A. N. Severtsov Institute of Ecology and Evolution, 119071 Moscow, Leninsky Prospect 33, Russian Federation; 29 FGBU Zapovednoye Priamurye, Komsomolskiy Department, 681000 Khabarovskyi krai Komsomolsk-on-Amur, Mira avenue, 54, Russian Federation; 30 Tigirek State Nature Reserve, 656043 Barnaul, Nikitina street 111, Russian Federation; 31 Institute of Systematics and Ecology of Animals of Siberian Branch of Russian Academy of Science, 930091 Novosibirsk, Frunze 11, Russian Federation; 32 State Nature Reserve Bolshaya Kokshaga, 424038 Mary El Republic Yoshkar-Ola, Voinov-Internacionalistov 26, Russian Federation; 33 Institute of Plant and Animal Ecology, Ural Branch, Russian Academy of Sciences, 620100 Ekaterinburg, 8 Marta 202/3, Russian Federation; 34 Sikhote-Alin State Nature Biosphere Reserve named after K. G. Abramov, 692150 Primorsky krai Terney, Partizanskaya 44, Russian Federation; 35 FSBI Prioksko-Terrasniy State Reserve, 142200 Moscow region Serpukhov district, Danky, Russian Federation; 36 National park Meshchera, 601501 Vladimir region Gus-Hrustalnyi, Internacionalnaya 111, Russian Federation; 37 Ilmensky State Nature Reserve, Russian Academy of Sciences, Urals Branch, 456317 Chelyabinskaya oblast Miass, Russian Federation; 38 FGBU National Park Kenozersky, 163000 Arkhangelsk, Embankment of the Northern Dvina, 78, Russian Federation; 39 FGBU GPZ Kologrivskij les im. M.G. Sinicina, 157440 Kostromskaja oblast’ Kologriv, Nekrasova 48, Russian Federation; 40 Altai State University, 656049 Lenin Ave. 61, Barnaul, Russian Federation; 41 Pryazovskyi National Nature Park, 72312 Zaporiz’ka oblast Melitopol, Interkulturna Street, 21/1, Ukraine; 42 State Nature Reserve Privolzhskaya Lesostep, 440031 Penza, Okruzhnaya 12-a, Russian Federation; 43 Komarov Botanical Institute of the Russian Academy of Sciences (BIN RAS), 197376 Saint Petersburg, Professora Popova 2, Russian Federation; 44 Sary-Chelek State Nature Reserve, 715705 Dzalal-Abad region, Aksu district, Arkyt village, Kyrgyzstan; 45 Institute for Evolutionary Ecology NAS Ukraine, 03143 Kiev, Lebedeva 37, Ukraine; 46 FGBU State Nature Reserve Kuznetsk Alatau, 652888 Kemerovo region, Mezhdurechensk, Shakhterev 33-1, Russian Federation; 47 Kerzhenskiy State Nature Biosphere Reserve, 603001 Nizhny Novgorod, Rozhdestvenskaya 23, Russian Federation; 48 Bryansk Forest Nature Reserve, 242180 Bryansk region Suzemka district, Nerussa St., Zapovednaya street, 2, Russian Federation; 49 Pinezhsky State Nature Reserve, 164610 Arhangel region Pinezkiy district, Pinega, Pervomayskaya street, 123 А, Russian Federation; 50 The Central Chernozem State Biosphere Nature Reserve named after Professor V.V. Alyokhin, 305528 Kurskiy region Kurskiy district, p/o Zapovednoe, Russian Federation; 51 Tyumen State University, 625043 Tyumen, Pirogova str., 3, Russian Federation; 52 Reserves of Taimyr, 666300 Norilsk, str. Talnakhskaya, entrance 2, Russian Federation; 53 Chatkalski National Park, 100059 Toshkent, Shota Rustaveli St., 144-34, Uzbekistan; 54 National Park Ugra, 248007 Kaluga, Prigorodnoe lesnichestvo, 3a, Russian Federation; 55 Kaniv Nature Reserve, 19000 Kaniv, Shevchenko str. 108, Ukraine; 56 Smolenskoe Poozerje National Park, 216270 Smolensk Region Demidovskiy district, Przhevalskoe, Gurevitch street 19, Russian Federation; 57 FSBI Zeya State Nature Reserve, 676246 Stroitelnaya str. 71, Zeya, Amurskaya Oblast Russian Federation; 58 Polistovsky State Nature Reserve, 182840 Pskov region Bezhanitsy district, Bezhanitsy Sovetskaya street, 9B, Russian Federation; 59 Ural State Pedagogical University, 620017 Yekaterinburg, prosp. Kosmonavtov, 26, Russian Federation; 60 Institute of Mathematical Problems of Biology RAS – the Branch of the Keldysh Institute of Applied Mathematics of Russian Academy of Sciences, 142290 Moscow Region Pushchino, Prof. Vitkevicha 1, Russian Federation; 61 Kronotsky Federal Nature Biosphere Reserve, 684000 Kamchatka region Yelizovo, Ryabikova street 48, Russian Federation; 62 Zhiguli Nature Reserve, 445362 Samara region, P. Bakhilova Polyana, Zhigulyovskaya 1, Russian Federation; 63 Institute for Ecology and Geography, Siberian Federal University, 660041 Krasnoyarsk, 79 Svobodny pr., Russian Federation; 64 Central Forest State Nature Biosphere Reserve, 172521 Tver region Nelidovo district, Zapovedniy village, Russian Federation; 65 National Park Bashkirija, 453870 Bashkortostan Republic Meleuzovskiy district, Nurgush, Abubakirova 1, Russian Federation; 66 State Nature Reserve Kurilsky, 694500 Sakhalin Juzhno-Kurilsk, Zarechnaya 5, Russian Federation; 67 Vodlozersky National Park, 185002 Karelia Petrozavodsk, Parkovaya 44, Russian Federation; 68 State Nature Reserve Kivach, 186220 Kondopoga District, Republic of Karelia Russian Federation; 69 South-Ural Federal University, 4563304 Chelyabinskaya oblast Miass, ul. Kalinina 37, Russian Federation; 70 Saint-Petersburg State Forest Technical University, 194021 St. Petersburg, Institutsky per. 5, 1-338-3, Russian Federation; 71 Astrakhan Biosphere Reserve, 414021 Astrakhan, Tsaerv River Bank 119, Russian Federation; 72 FSBI United Administration of the Lazovsky State Reserve and national park Zov Tigra, 692980 Primorskiy Krai Lazovskiy District, Lazo, Centralnaya, 56, Russian Federation; 73 State Nature Reserve Tungusskiy, 660028 Krasnoyarsk region Krasnoyarsk, Street 27 19, Russian Federation; 74 Krasnoyarsk State Pedagogical University named after V.P. Astafyev, 660049 Krasnoyarsk, Ada Lebedeva st. 89, Russian Federation; 75 Institute of Geography, Russian Academy of Sciences, 119017 Moscow, Staromonetniy 29, Russian Federation; 76 Koltzov Institute of Developmental Biology, Russian Academy of Sciences, 119334 Moscow, Vavilov Street 26, Russian Federation; 77 Carpathian National Nature Park, 78500 Ivano-Frankivsk region Yaremche, V. Stusa street 6, Ukraine; 78 State Environmental Institution National Park Braslav lakes, 211970 Vitebsk region Braslav, Dachnaya 1, Belarus; 79 National Park Synevyr, 90041 Zakarpattia Region Mizhhirs’kyi district, Synevyr-Ostriki, Ukraine; 80 Pasvik State Nature Reserve, 184421 Murmansk region Nikel, Gvardeyskiy Ave. 43, Russian Federation; 81 Mari Chodra National Park, 425090 Mari El Republic Zvenigovsky District, Krasnogorsky Settlement, Tsentralnaya Street, 73, Russian Federation; 82 Information-Analytical Centre for Protected Areas, 123242 Moscow, Kapranova side-street 3, Russian Federation; 83 State Nature Reserve Vishersky, 618590 Perm region Krasnovishersk, Gagarina street 36B, Russian Federation; 84 State Nature Reserve Olekminsky, 678100 Republic Sakha Olekminsk, Filatova 6, Russian Federation; 85 Crimea Nature Reserve, 298514 Alushta, Partizanskaya, 42, Republic of Crimea; 86 Forest Research Institute Karelian Research Centre Russian Academy of Sciences, 185910 Karelia Petrozavodsk, Pushkinskaya 11, Russian Federation; 87 Black Sea Biosphere Reserve, 75600 Khersons’ka oblast Hola Prystan, Mikhail Lermontov 1, Ukraine; 88 Institute of Physicochemical and Biological Problems in Soil Sciences, Russian Academy of Science, 142290 Moscow Region Pushchino, Institutskaya 2, Russian Federation; 89 State Nature Reserve Nurgush, 610002 Kirov, Lenina street, 129a, Russian Federation; 90 Caucasian State Biosphere Reserve of the Ministry of Natural Resources, 385000 Adygea Republik Maykop, Sovetskaya str. 187, Russian Federation; 91 National Nature Park Vyzhnytskiy, 59200 Chernivtsi Region Vyzhnytsya District, Berehomet, Street Central 27 а, Ukraine; 92 National Park Khvalynsky, 412780 Region Saratov Khvalynsk Sity, Oktyberskya Street, 2b, Russian Federation; 93 State Research Center Arctic and Antarctic Research Institute, 199397 Saint Petersburg, Bering st. 38, Russian Federation; 94 Mordovia State Nature Reserve, 431230 Mordovia Republic Temnikov region, village Pushta, Russian Federation; 95 State Nature Reserve Malaya Sosva, 628242 Tjumen region Sovetskiy, Lenina str., 46, Russian Federation; 96 Surhanskiy State Nature Reserve, 191404 Surhandarja region Sherabad, Agahi, 1, Uzbekistan

Correction to: *Scientific Data* 10.1038/s41597-020-0376-z, published online 11 February 2020

*Following publication it was found that co-author Aleksandr Ananin’s second affiliation was missing. The additional affiliation has now been added to both the HTML and PDF versions of the Data Descriptor*.

